# Biopolymer-Based Nanosystems for siRNA Drug Delivery to Solid Tumors including Breast Cancer

**DOI:** 10.3390/pharmaceutics15010153

**Published:** 2023-01-01

**Authors:** Md Abdus Subhan, Vladimir P. Torchilin

**Affiliations:** 1 Department of Chemistry, ShahJalal University of Science and Technology, Sylhet 3114, Bangladesh; 2 CPBN, Department of Pharmaceutical Sciences, North Eastern University, Boston, MA 02115, USA; 3 Department of Chemical Engineering, North Eastern University, Boston, MA 02115, USA

**Keywords:** biopolymer, drug delivery, solid tumors, breast cancer, siRNA therapy

## Abstract

Nanobiopolymers such as chitosan, gelatin, hyaluronic acid, polyglutamic acid, lipids, peptides, exosomes, etc., delivery systems have prospects to help overwhelmed physiological difficulties allied with the delivery of siRNA drugs to solid tumors, including breast cancer cells. Nanobiopolymers have favorable stimuli-responsive properties and therefore can be utilized to improve siRNA delivery platforms to undruggable MDR metastatic cancer cells. These biopolymeric siRNA drugs can shield drugs from pH degradation, extracellular trafficking, and nontargeted binding sites and are consequently suitable for drug internalization in a controlled-release fashion. In this review, the utilization of numerous biopolymeric compounds such as siRNA drug delivery systems for MDR solid tumors, including breast cancers, will be discussed.

## 1. Introduction

Biopolymers are promising to apply for drug delivery as well as therapeutic agents for cancer therapy. Biopolymeric materials demonstrate enhanced biocompatibility as well as siRNA delivery competence, targeting varieties of cancer genes in solid tumors in vitro and in vivo. Both natural and synthetic biopolymers are effective vehicles for siRNA drug delivery to cancer cells. Biopolymers, such as alginate, pullulan, cellulose, polylactic acid, chitosan, and exosomes have been efficiently utilized in siRNA drug delivery to solid tumors. Further, siRNA therapeutics have been meticulously explored and have been translated to the clinic through lipid NPs [1,2]. However, there are several pitfalls in siRNA delivery proficiency and targeting. Cationic lipids have toxicity since they can interact with enzymes [3,4]. Cationic lipids can interact with RNA and enable encapsulation; however, they can also be toxic [5]. A high concentration of cationic lipids can destroy the cellular membrane, causing cell lysis and necrotic death [3,4]. To overcome these shortcomings, different polymer-based nanoparticle carriers such as biopolymers, exosomes, and peptide nanoassemblies have been utilized for efficient siRNA delivery approaches [6]. Exosomes are extracellular vesicles, and they are mediators of short- and long-distance intercellular communication in health and diseases. Exosomes are promising platforms for siRNA delivery due to their nonimmunogenic and nontoxic natures. Exosomes can be extracted from different biological sources, such as cells and tissues.

Many natural polysaccharide polymers are utilized in siRNA delivery [7]. The key advantage of polysaccharides is the presence of various functional groups suitable for functionalization to achieve structural variety and polymers [8]. The most frequently utilized polysaccharide for siRNA delivery is chitosan. The primary amino groups in chitosan make it a polycation that facilitates the association of siRNA and complex formation [9]. For increasing the solubility of chitosan, numerous alterations have been performed, and water-soluble chitololigosaccarides have been fabricated. These chitololigosaccarides have been utilized as siRNA delivery vehicles [10]. 

Collagen is another biocompatible, safe natural biopolymer, which is suitable for drug delivery. For one study, siRNA-loaded collagen formulations were fabricated and utilized as localized and sustained-released platforms of siRNA in vivo to solid tumors with promising outcomes [7,11].

Biopolymers such as HA or chitosan are utilized in drug delivery as siRNA vehicles. These biomaterials are manageable via numerous alterations. PEI, aminopropyl triethoxysilane (APTES), and chitosan are cationic polymers used for coating nanoparticles for packing siRNA through electrostatic attractions [12,13,14]. APTES is frequently utilized for amine functionalization of mesoporous silica nanoparticles (MSNs) [14]. Chitosan is an amine-rich natural polysaccharide, which is an excellent biopolymer for siRNA delivery [15]. Several anticancer drugs have been delivered by the chitosan–MSN system to tumors, including breast tumors [16,17,18]. One study reported on chitosan-functionalized MSN for gene delivery with EPR (enhanced permeability and retention) uptake in HeLa cells [17]. 

Chitosan acts as an siRNA delivery vehicle due to its exclusive benefits, cationic charge, and simple alteration [1,19]. The key drawback of using chitosan-based polymers as cancer drug carriers is the low transfection competence and endosomal release. Chitosan-based polymeric NPs for siRNA delivery demonstrated improved transfection efficiencies by controlling some features such as length, charge, etc. [20]. To enhance the transfection efficiency of chitosan NPs, an ionic gelatin method has been developed [21]. The incorporation of heparin into chitosan nanoparticles increased transfection efficiency. The efficiency of siRNA-induced silencing of VEGF of ARPE-19 cells was 25% more with heparin compared to chitosan nanoparticles without heparin [22].

Recently, the US-FDA has approved four siRNA-based drugs: patisiran, givosiran, lumasiran, and inclisiran. For this reason, siRNA drugs have received increased attention of researchers. Biopolymers are auspicious as siRNA drug delivery platforms as well as therapeutic agents for effective cancer therapy.

## 2. Sources of Biopolymers Utilized as Drug Delivery Vehicles

**Figure 1:**
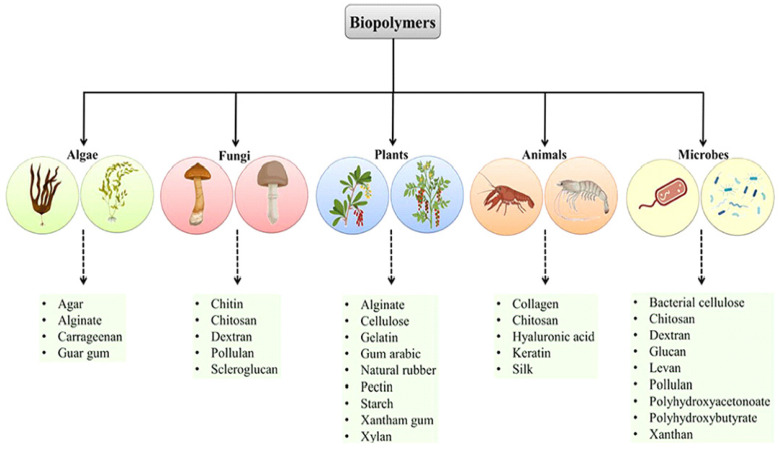
Biopolymers could be achieved for different sources. Adapted with permission from Ref. [23]. Copyright © 2023 Pathak, Singh, Singh, Sharma, Singh, Gupta, Mishra, Mishra and Tripathi.

Biopolymers or green polymeric materials are promising to apply in drug delivery as well as therapeutic agents for cancer therapy [23,24]. Biopolymers including alginate, pullulan, cellulose, polylactic acid, chitosan, and exosomes have been efficiently utilized in siRNA drug delivery to cancer cells. These biopolymers can be achieved from different sources such as algae, fungi, plants, animals, microbes, etc. (Figure 1). However, exosomes are obtained from tissues, biological fluids, and conditioned media of cells in culture. Biopolymers have gained a lot of attention in the advancement of innovative anticancer drug delivery platforms. Biopolymer-based NPs can be developed in many ways. These biopolymeric NPs have been established as suitable platforms for efficient drug delivery with enhanced selectivity and low toxicity. 

Biopolymers are usually composed of non-toxic, safe monomers, and they are carbon neutral for the environment. Biomass morphology is generally structured as nanomaterials. As a result, biopolymers enable the synthesis of biocompatible nanomaterials [23,24]. Chitosan, starch, dextran, and cellulose are polysaccharides derived from plants and microbial biofilms; these biopolymers are frequently utilized for the synthesis of nanomaterials. Biopolymers are unique in composition and possess various important physico-chemical and physiological properties suitable for drug delivery. Further, biopolymeric nanomaterials can be formed by attaching metals, metal oxides, and metal sulfides to biopolymers [25,26]. Biopolymers can form molecular capsules by intramolecular hydrogen bonding. For example, starch can form polymeric nanocomposites by incorporating metals or metal oxides. Further, chitosan and derivatives are frequently used for producing biocompatible nanoparticles for drug delivery. Nanomaterials can be incorporated in biopolymers by both sorption and impregnation [23,24,27]. 

## 3. Biopolymer-Based Nanocarrier for siRNA Drug Delivery to Solid Tumors 

The importance of effective cancer therapy is emerging increasingly [28]. Due to the feasibility of polymer functionalization, enhanced targeting effects have been observed with polymeric NPs decorated with different peptides or ligands. Chitosan-based NPs were conjugated with HAD (hyaluronic acid dialdehyde) to fabricate an siRNA nanoplatform, siRNA@chitosan-HAD, for the delivery of siRNA targeting CD44 [29]. A natural biopolymer, curdlan, was modified, and represented as CuVa polymers it demonstrated enhanced biocompatibility as well as siRNA delivery competence targeting the *STAT3* gene in solid tumors in vitro [30]. Exosomes result from extracellular vesicles formed in the cells [31]. They are prospective candidates for siRNA delivery, as they are easy to handle, nonimmunogenic, and nontoxic [32,33]. Bovine milk exosomes are used as vehicles for siRNA delivery to tumors. siRNA delivery via milk exosomes silenced major oncogenes with 2- to 10-fold knockdown in several solid cancers in vitro and in vivo [33]. Exosomes with a targeted approach (e.g., CD47 targeting) have shown promising outcomes in solid tumors of deadly cancers, including GBM (glioblastoma), PDAC (pancreatic ductal adenocarcinoma), and TNBC (triple-negative breast cancer) [33,34,35]. The angiopep-2 (An2)-functionalized STST3siRNA-loaded exosome (Exo-An2-siRNA) is a potential drug to enhance the treatment of solid tumors. Exo-An2-siRNA demonstrated excellent blood stability, efficient cellular uptake, and effective BBB penetration ability in GBM patients. Exo-An2-siRNA exhibited enhanced in vitro anti-cancer effects, as exosomes protect siRNA, and modified An2 targets tumors effectively. Exo-An2-siRNA showed inhibition of proliferation in a xenograft model of GBM with no side effects and enhancing median survival time. The developed nanoplatform Exo-An2-siRNA is a promising, safe therapy approach for solid tumors targeting the *STAT3* gene. This approach may be effective in breast cancer therapy [35].

A method was developed to utilize serum-derived exosomes as drug delivery carriers for siRNA drugs in vivo. The study used exosomes as vehicles for siRNA delivery to lung macrophages in vivo; exosomes containing siRNA exerted functional roles in the lung in vivo. The serum-derived exosomes are non-toxic and do not activate immune responses and were found to be safe for in vivo use [36]. 

Further, bovine milk exosomes were utilized as an innovative siRNA delivery platform targeting specific genes including VEGF, EGFR, AKT, MAPK, BCl2, SUR, and KRAS [33]. A 5′^−32^ P-labeled siKRAS was used as a tracer for evaluating exosome loading with siRNAs. The study exhibited the knockdown of target gene expression ranging from 2- to 10-fold in different cancers by exosome-loaded siRNAs. Since KRAS has been correlated with different solid cancers, siKRAS delivery to different solid tumors may be a promising siRNA therapeutic approach for solid tumor therapy. In this study, siKRASG12S was used to target KRAS in lung cancer cells. Extracellular vehicles, particularly exosomes derived from milk used as carriers for siRNA-loading and delivery signify a feasible natural nanocarrier for siRNA delivery to cancer cells utilizing systemic or oral delivery platforms [33,37,38]. 

The cellular membrane (~5 nm) can be passed by cell-penetrating peptides (CPPs), 5–30 residue peptides [39]. Peptides are important siRNA drug carriers. In particular, CPPs are short cationic peptides that facilitate intracellular delivery of several biological cargos, including siRNA and mRNA [40]. 

Amphiphilic phospholipid peptide dendrimers (AmPPDs) were fabricated as an efficient siRNA delivery platform. A DSPE-KK_2_-AmPPDsiRNA delivery system mediated the effective intracellular uptake and endosomal release of siRNA complexes and resulted in gene knockdown, demonstrating anticancer efficiency both in vitro and in vivo in solid tumors [41]. A peptide-based p5RHH NPs-condensing oligonucleotide was developed for siRNA delivery, which reduced the KRAS gene in solid tumors in vivo, demonstrating cancer inhibition effectively [42]. 

Introduction of protein and mAbs into a polymeric vehicle can advance the siRNA delivery to tumors [43,44,45]. A nanosystem composed of a cyclodextrin-containing polycation, an adamantane-conjugated PEG (AD-PEG), human transferrin-decorated AD-PEG, and therapeutic siRNA was fabricated. The hTf receptor is overexpressed on tumor cells. The developed nanosystem successfully delivered siRNA to tumor cells through hTf receptor-mediated endocytosis, and it downregulated the target genes in solid tumors. The nanocaplet transferred siRNA into the cytoplasm and caused gene silencing through RNAi. The targeted NPs also showed deep tissue penetration due to transferrin-stimulated transcytosis [46]. A 3D spheroid study with Hep3B cancer cells exhibited a transferrin conjugated nanosystem delivered siRNA effectively into the spheroid with a depth of 70 μm, indicating Tf-mediated deep tissue permeation, gene knockdown, and cancer cell inhibition [43,46]. In this study, the deep tissue penetration of the nanocaplet was evaluated by confocal laser scanning microscopy (CLSM, λ_ext_ = 488) using siRNA^A488^ fluorescently labeled with Alexa Flour 488 in the nanodrugs. Since the brain endothelial cells overexpress Tf-receptors, the nanocaplet may have the potential to overcome the blood–brain barrier. Thus, an in vivo study utilizing the nanocaplet may be suitable for delivering siRNA to brain tissues, such as GBM. 

Poly-L-arginine and siRNA were consecutively adsorbed on an anionic liposome NP, and the outermost layer was decorated with propargyl-modified NPs with 1,2-distearoyl-sn-glycero-3-phosphoethanolamine (DSPE), DSPE-PEG, and cholesterol to fabricate long blood-circulating materials [43,47]. Such coformulation of cationic polymers with lipid improved the polyplex stability and enhanced siRNA loading efficacy and steadiness [48,49,50]. 

**Figure 2:**
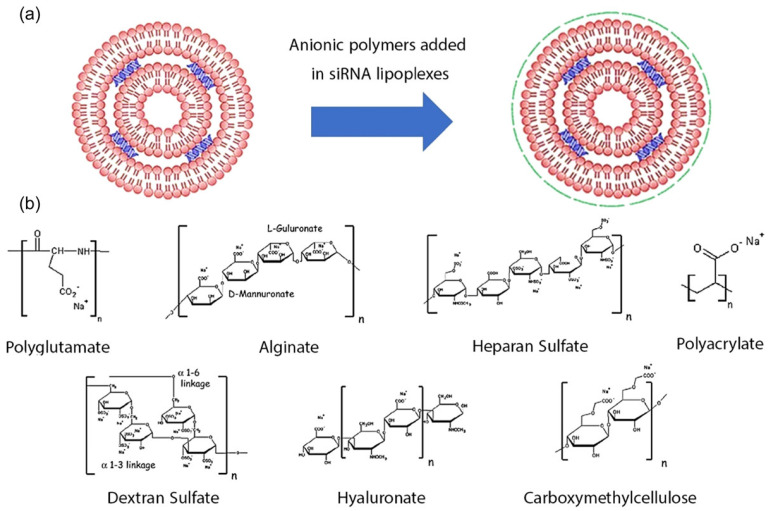
(**a**) Anionic polymers included in siRNA lipoplexes. (**b**) Different anionic polymers. Adapted with permission from Ref. [21]. Copyright © 2023–2022 John Wiley & Sons, Inc.

siRNA–lipid complexes are known as lipoplexes. Cationic liposomes combined with lipids have shown excellent outcomes in siRNA drug delivery for cancer therapy [21]. However, immune responses and undesired toxicities associated with lipoplexes should be addressed. Various approaches have been developed to diminish lipoplex toxicity. Although their entrapment efficiency might be low, neutral liposomes have reduced such toxicity. Further, cationic lipoplexes have been coated with anionic polymers, including sodium salts of hyaluronic acid, and alginic acid carboxymethyl cellulose. The coated cationic liposomes showed enhanced siRNA delivery to solid tumors (Figure 2) [21]. 

The benefit of polymeric nanocarriers is that they are not stimulators of the immune response. Electrostatic interactions between polymers and siRNA are readily achievable through linkages (thiol, maleimide, disulfide, etc.). The natural cationic polymers have led to efficient delivery approaches due to low toxicity, biodegradability, and biocompatibility [21,51,52,53]. 

The siRNA rigid structure incites weak interactions with polycation, and polyplexes are less effective in protecting siRNA against nucleases. Increasing the polycation amount could improve this pitfall while increasing toxicity, and hence an optimum polycation quantity should be utilized. Further, dendrimers have flexibility and prospects in siRNA loading [21,54]. 

Nanoparticles PEG-*b*-PLA or PEG-*b*-PLGA have received increased curiosity for siRNA drug delivery but are restricted by the reduced encapsulation efficacy of siRNA. To overcome this pitfall, a cationic lipid-induced NP platform known as CLAN with more siRNA encapsulation competence was fabricated [55].

Cell membrane coating has been established as an efficient approach to enhance the in vivo execution of NPs. Cell membrane decoration on NPs improves their colloidal stability and blood circulation and also benefits targeted drug delivery. Cancer cell membrane-decorated biomimetic NPs could deliver siRNA to homologous tumors in vivo [56,57]. Cell membrane-coated polyplexes are associated with problems in releasing siRNA into cytoplasm. To resolve this problem, charge-reversal polymers are utilized to facilitate siRNA release in cytoplasm [58,59]. PEI–siRNA polyplexes were coated with citraconic anhydride-grafted poly-L-lysine, which was then decorated with angiopep-2 peptide-coated red blood cell membrane (Ang-RBCm) [59]. The RBC membrane decoration offered long-term blood circulation, and targeting of angiopep-2 enhanced the accumulation of drugs into solid tumors. Such cell membrane-decorated charge reversal hybrid nanocomplexes proficiently inhibited tumor growth by knockdown of target genes via siRNA delivery.

Virus/polymer chimeric NPs permit simultaneous expression and silencing of numerous genes [60,61]. NPs comprising a Bcl-2-interacting mediator of cell death (BIM)-encoding adeno-associated virus (AAV) core and an acid-degradable polyketal (PK) shell were developed for enhanced siRNA delivery to tumors. After endocytosis of nanoparticles into the cell, the release of siRNA was facilitated by PK shell degradation. The BIM-encoding AAV was then transported into the cell nucleus for BIM expression. BIM enhanced the apoptosis of cancer cells. Thus, the polymer/virus chimeric NPs upregulated the BIM expression and simultaneously inhibited the cancer cells [43,60,61]. 

PLL produced by *Streptomyces albus* was utilized as a scaffold for the production of polyphenol-grafted bio-inspired polymers for siRNA delivery. Phenol, catechol, and pyrogallol were conjugated onto PLL through a simple reaction, and the cellular uptake of polyphenol-grafted polymer-siRNA complex by HeLa cells was examined [62]. A series of polyphenol-grafted low molecular weight polymer–siRNA complexes was effective in siRNA delivery inspired by natural polyphenol. The phenolic moieties enhanced the siRNA binding ability and stability of the polyplex. Among the polyphenols, polycatechols demonstrated the highest performance in siRNA binding, cellular uptake, and gene silencing [62]. The polycatechol–siRNA platform could be a highly promising bioinspired polymer for siRNA delivery in solid tumors. 

**Figure 3:**
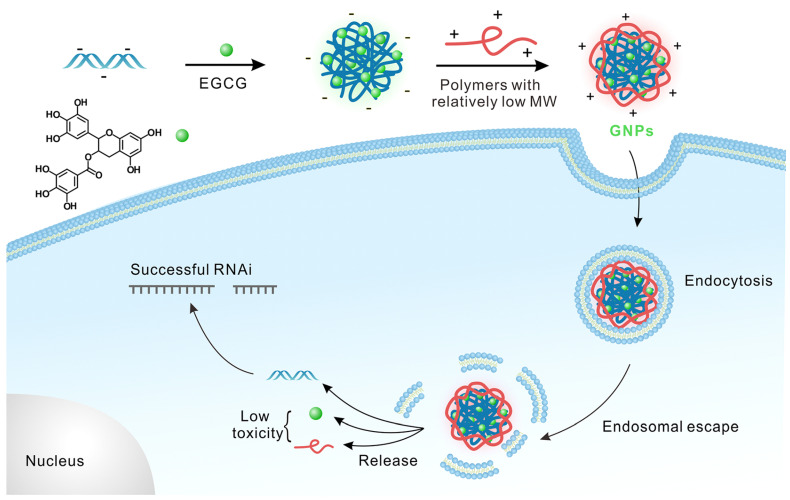
GNPs prepared by the supramolecular approach exhibited a gene silencing mechanism with high efficiency and low toxicity. Adapted with permission from Ref. [63]. Copyright © 2023 American Chemical Society.

Green tea-based EGCG enables siRNA condensation by low molecular-weight polymers, yielding green NPs (GNPs), showing effective siRNA-induced gene silencing [63]. Biocompatible components of GNPs such as EGCG, siRNA, and PLL demonstrated reduced toxicity on the transfected cells. Similarly, antisense oligodeoxynucleotides, DNAzymes, and peptide nucleic acids may be utilized to design bioinspired polymer-based platforms for the fabrication of GNPs for a robust system that is promising for gene/siRNA delivery (Figure 3) [63].

Further, utilizing siRNA drugs for the treatment of hematological malignancies has been ineffective because blood cancer cells demonstrate significant resistance to standard transfection methods [64]. However, there are some studies concerning siRNA delivery to blood cancers. For example, CD20/CD44 dual targeted siRNA delivery using layer-by-layer NPs (LbL NPs, PLGA/PLA/siRNA/PLA; PLGA, poly-(D,L-lactide-co-glycolide); PLA, poly-l-arginine) with BCL-2 siRNA in B cell lymphoma and leukemia cells downregulated *BCL-2*, induced apoptosis, and reduced the blood cancer cells. Herein, the outermost layer of LbL NPs consists of hyaluronic acid (a CD44-ligand) covalently conjugated to CD20 antibodies. The CD20/CD44 dual-targeting outer layer offered precise binding to blood cancer cells, followed by receptor-mediated endocytosis of the LbL NPs [64]. 

In recent years, there have been many pre-clinical studies using biopolymer-induced siRNA therapeutics for the enhanced therapy of cancer. To date, several siRNA-mediated drugs have been in different phases of clinical trials or have received FDA approval for the treatment of a variety of diseases, including solid tumors, as demonstrated in Table 1 [65,66]. siRNA drugs including patisiran, givosiran, lumasiran, and inclisiran have received approval from the FDA [67]. As a result, siRNA drugs have received the enhanced attention of medical doctors and scientists. The design of apposite siRNA-assisted delivery systems is a vital issue for clinical applications in cancer therapy. These approaches could be potentially supportive in reducing cancer deaths in the near future.

## 4. Breast Cancer Therapy with Biopolymer-Based siRNA Nanoplatform

Breast cancer is the second leading cause of death in woman after lung cancer world-wide. However, cancer death rates including breast cancer have been decreasing due to the development of advanced screening technologies and significant improvement of immunotherapy, targeted therapy, and gene therapy [68]. Natural herbal compounds or biopolymers have significant roles in inhibiting drug resistance in breast cancer, impeding breast cancer stem cells (BCSCs). BCSCs utilize different cellular mechanisms, including membrane transporters, anti-apoptotic, pro-survival, and self-renewal-linked signaling pathways. Biopolymers may disrupt these mechanisms and hence impede breast tumors, inhibiting the BCSCs, and the induction of apoptosis [69]. Therefore, this review highlighted developments in biopolymer-mediated siRNA drug delivery systems for breast tumors. Biopolymer-induced delivery of chemotherapeutics and siRNA is promising and has demonstrated synergistic effects as a single delivery therapeutic [12]. Methotrexate (MTX) is a potential drug for breast cancer treatment, effectively inhibiting the dihydrofolate reductase (DHFR) receptor, which suppresses DNA synthesis. MTX can significantly induce apoptosis by inhibiting the STAT3 in the JAK/STAT pathway in tumor cells. Inhibition of the STAT3 protein by siRNA is an effective approach to hinder breast tumors [70]. A nanoparticle delivery system for the co-delivery of MTX and STAT3siRNA was developed and investigated against MCF-7 breast cancer cells in vitro. Mesoporous silica nanoparticles (MSNs) were functionalized with chitosan by covalent grafting facilitated by APTES using glutaraldehyde as a linker. APTES and grafted chitosan offered an amine rich surface coating on MSN NPs, permitting electrostatic loading of both MTX and siRNA on MSN [14]. The co-delivery of MTX and STAT3siRNA to breast cancer cells in vitro efficiently inhibited the DHFR receptor and inhibited breast tumors. Further, the co-delivery of doxorubicin (Dox) and p53 by deionized chitosan derivative functionalized MSNs tempted noteworthy apoptosis in breast cancer cells due to the synergistic effects of Dox and the p53 gene [18]. 

PEG-modified chitosan was synthesized for survivin-targeted siRNA delivery to breast cancer cells to inhibit breast tumors and to prevent metastasis [71]. The outcome of the study demonstrated that siRNA loaded in PEGylated chitosan (PEG-chitosan-siRNA) delivered to breast cancer cells showed enhanced cellular uptake and inhibited 4T1 tumor cell growth significantly. The flow cytometry study and confocal scanning microscopy study demonstrated that PEG-chitosan-siRNA particles were uptaken more easily than naked siRNA. The developed PEG-chitosan-siRNA nanoparticle significantly reduced tumor growth in vivo in a xenograft mouse model. The study exhibited that the PEG-chitosan-siRNA nanoplatform is safe, effective in tumor inhibition, and efficient in preventing metastasis [71].

Gold NPs modified with trimethyl chitosan (TMC) were developed as efficient delivery carriers to enhance the stability and cellular uptake of siRNA targeting EGFR in breast tumors. EGFRsiRNA was complexed with AuNPs-TMC through electrostatic interactions to develop the nanosystem AuNPs-TMC-EGFRsiRNA using a facile synthesis process. The synthesized AuNPs-TMC-EGFRsiRNA NPs effectively delivered siRNA to breast cancer cells. Therefore, AuNPs-TMC-EGFRsiRNA appeared as a prospective and potential nanodelivery system for breast cancer therapy [72]. 

A breast cancer-specific gene 1-siRNA (BCSG1-siRNA) plasmid was fabricated and then encapsulated by chitosan-silicon dioxide NPs. The slow release of BCSG1-siRNA to MCF-7 cells demonstrated enhanced cytotoxicity, reduced proliferation, and increased apoptosis. BCSG1-siRNA downregulated BCSG1 and inhibited tumor growth significantly. As a result, chitosan-silicon dioxide NP-BCSG1-siRNA is an effective siRNA delivery system to inhibit breast tumors [73]. 

A pH-responsive nanoplatform was fabricated introducing antiPOL siRNA and guanidine-CO_2_-functionalized chitosan at a neutral pH to knock down the POLR2 in TNBC tumors. The siRNA nanodrug was effectively internalized into TNBC cells harboring hemizygous TP53 deletion, overcoming intracellular trafficking at low pH. The downregulation of POLR2 caused TNBC cell death with little or no toxicity [74].

A triblock copolymer, a hollow-sphere polymersome PVPON-PDMS-PVPON was designed for siRNA delivery to breast cancer cell lines [75]. The polymersomes assembled in the presence of 1 μM PARPsiRNA-loaded siRNA inside the nanocarrier. When the developed nanovehicles were disrupted by ultrasound in vitro, the siRNA was released unaltered. siRNA-loaded polymersomes were tested against HER2+, trastuzumumab-resistant breast cancer cells in vitro, which reduced the PPARP1 protein level in the breast cancer cells and impeded MDR genes. Herein, a fluorescent dye was attached covalently to the outside of the nanocapsules. Thus, the targeting molecule was added to the nanocapsules to deliver with the polymersomes into the tumors. This nanosystem with PARP1siRNA was treated with TNBC cells in a tumor mouse model, and the delivered siRNA significantly knocked down the expression of PARP1 and increased by four-fold the survival of mice bearing TNBC tumors. PARPis are suitable for targeting TNBC tumors with defects in DNA repair and could alter the TME (tumor microenvironment) [76,77,78,79,80,81,82,83]. However, it has been challenging to combine many PAPRis with chemotherapy due to bone marrow suppression. Targeting PARP1i in the tumor may permit novel combination therapy approach. The polymersome-loaded PARP1siRNA nanosystem is a biodegradable, non-ionic polymeric nanovesicles proficient to encapsulate and deliver PARP1siRNA for knocking down PARP1 in vivo. This study provided an advanced nanopodium for the development of an effective drug delivery nanocarrier system for siRNA therapy of breast cancer, including TNBC [75]. 

A stable polyplex was developed using PEG-modified L-arginine oligo(-alkylaminosiloxane) grafted with PEI for siRNA delivery to breast cancer cells [84]. The polyplex was used to deliver the anti-luciferase siRNA to the luciferase-expressed TNBC cell line MDA-MB-231-Luc-D3H2LN, and anti-ABCB1 siRNA to doxorubicin-resistant MCF-7 ADR. Transfection of MDA-MB-231-Luc-D3H2LN cells with anti-luciferase siRNA demonstrated comprehensive knockdown of luciferase expression. When MCF-7 ADR cells were transfected with anti-ABCB1 siRNA, efficient downregulation of ABCB1 was observed. Therefore, this approach could be effective against breast cancer and TNBC therapy [84]. 

Lipid-based carriers are efficient in siRNA delivery [85]. Piperazine is frequently utilized in bioactive compounds and medicine [86,87]. A recent study reported that piperazine-containing ionizable lipid (Pi-Lipids) favorably deliver genes to immune cells in vivo without targeting the ligand [86]. Herein, the author demonstrated that Pi-A10 LNP (Pi-LNPs that contain PPZ-A10) effectively delivered mRNA/siRNA to solid tumors. LNP-10A was also formulated with siGFP and siLuc, and LNP10AsiGFP showed 25% knockdown of GFP, but no knockdown of luciferase was observed using LNP10AsiLuc. This study showed that high throughput in vivo investigations can recognize NPs with natural nonhepatocyte tropism, and the lipid with bioactive small molecule motifs can deliver mRNA/siRNA to immune cells in vivo. Further, LNP-10A was formulated with sipGP. This strategy could be applied to deliver siRNA to breast cancer cells [88]. 

CD44 expression is linked to the JAK2/STAT3 pathway in TNBC hostility. Incorporation of PEG–lipid complexes inside the lipid bilayers increased the sustained release of siRNA and evasion of the mononuclear phagocytic system. Cationic liposomes are widely used for siRNA delivery platforms for enhanced delivery, without enzymatic degradation, and effective cytoplasmic release [89]. 

Lipid-based polymeric nanocarriers are utilized for siRNA delivery to chemoresistant breast cancer. A nanoplatform formulated by hexadecylated PEI-siRNA encapsulated with lipid segments (DSPE-PEG, DSPE, cholesterol, tripalmitin) and fabricated with apolipoprotein was used for megalin-targeted siRNA delivery to chemo-treated breast cancer cells, which demonstrated enhanced delivery and a reduction of disease recurrence [89]. 

Calcium apatite NPs-siRNA downregulated BCL-2, EGFR, and ERBB-2 genes, resulting in the cell death-inhibiting PI3K/MAPK signaling pathway in a mouse model of breast tumors [90]. A calcium phosphate-PEG-polyanion polymer promoted the siRNA delivery targeting anti-apoptotic gene BCL-xL and BCL-2 in breast cancer cell line MCF-7. After 48 h of incubation the BCL-xL and BCL-2 genes were reduced by 49% and 23%, respectively [91]. 

Further, MCF-7 and MDA-MB-231 cell lines were studied to knock down SUR and BCL2 genes utilizing siSUR- and siBCL2-loaded bovine milk exosomes. siSUR was highly effective in inhibiting the breast cancer cells with ~80% knockdown of the SUR gene [33]. Bovine milk exosomes are natural, stable, safe, feasible, and promising nanocarriers for siRNA delivery to breast cancer cells.

NPs consisting of coupled PEI and folic acid to core/shell Fe_3_O_4_@SiO_2_ were fabricated to deliver Notch-shRNA to TNBC cell lines, MDA-MB-231 [92]. The study demonstrated that superparamagnetic Fe_3_O_4_@SiO_2_/PEI-FA/Notch-1shRNA NPs induced delivery of Notch-1shRNA to TNBC cells effectively with no toxicity. The nanodelivery cargo was capable of delivering Notch-1shRNA efficiently while protecting shRNA from degradation by exogenous DNaseI and nucleases. The preferential uptake of superparamagnetic Fe_3_O_4_@SiO_2_/PEI-FA/Notch-1shRNA NPs by TNBC cells MDA-MB-231 was revealed by MRI and fluorescence microscopy. Significantly decreased expression of Notch-1 was exhibited by MDA-MB-231 cells. Thus, the Fe_3_O_4_@SiO_2_/PEI-FA/Notch-1shRNA nanosystem is an effective theranostic system exploring both Notch-1 inhibition in TNBC cells and superparamagnetic property-enabled MRI and fluorescence imaging simultaneously [92]. 

A PEI nanocomplex was fabricated with EpCAM aptamer (EpApt) and EpCAM siRNA (siEP) to study its effects against breast cancer. The fabricated PEI-EpApt-siEp knocked down EpCAM and subdued the proliferation of breast cancer cells MCF-7. The study demonstrated that PEI-EpApt-siEp has higher binding ability to MCF-7 cells than EpApt or scrambled aptamer or PEI-ScrApt-SiEp [65,93].

A nanosystem was fabricated with lipid-NPs-siRNA coated with a Fab antibody to utilize against heparin-binding EGF-like growth factor (αHB-EGF LNP siRNA). A developed αHB-EGF LNP siRNA nanoplatform targeting PLK1 was examined for TNBC cell line MDA-MB-231 overexpressing HB-EGF on the cell surface. The result indicated the suppression of PLK1 protein, with the inhibition of the TNBC tumor revealing αHB-EGF LNP siRNA as a prospective target for TNBC tumors expressing HB-EGF [94].

A potential nanoplatform using a liposome–polycation–DNA complex (LPD) conjugated with tumor specific antibody (TLPD) was developed for siRNA delivery targeting EGFR in TNBC cells. TLPD was prepared by a self-assembled process followed by anti-EGFR Fab antibody conjugation (TLPD-FCC). The TLPD-FCC-siRNA demonstrated enhanced binding affinity and luciferase gene silencing (20%) in EGFR, overexpressing the TNBC cell line MDA-MB-231 in vitro. This study showed that TLPD-FCC has great prospects in delivering siRNA to EGFR overexpressing breast cancer cells [95].

A TNBC tumor targeting RGD-PEG-ECO/siDANCR nanosystem was developed for studying breast cancer cells. TNBC cell lines MDA-MB-231 and BT549 cells treated with the RGD-PEG-ECO/siDANCR NPs showed ~90% silencing of DANCR expression for up to 7 days, demonstrating an effective siRNA delivery approach for targeting and an enhanced therapeutic effect [96]. 

ICAM-1 targeted Lcn2 siRNA encapsulated liposome binds TNBC cells MDA-MB-231 significantly, and effective Lcn2 downregulation by ICAM-Lcn2-LPs led to a substantial lessening in VEGF from MDA-MB-231 cells, resulting in diminished angiogenesis, both in vitro and in vivo. The study demonstrated a promising approach for inhibiting angiogenesis and for tumor growth retardation in TNBC tumors [97]. 

**Figure 4:**
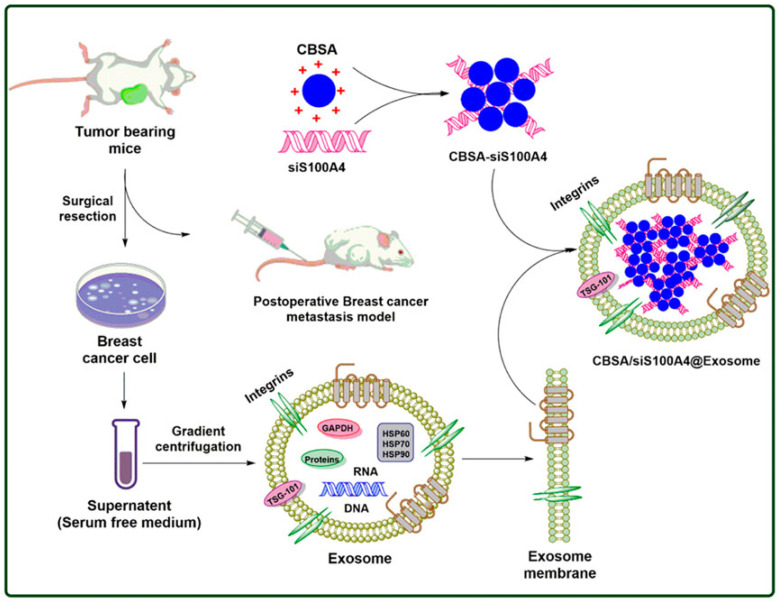
Post-operation metastatic inhibition in breast tumors through CBSA/siS100A4@Exosome. Adapted with permission from Ref. [99]. Copyright @2022, Goyal, Chopra, singh, Dua and Gautam.

Cationic bovine serum albumin-conjugated siS100A4 was coated with exosome membrane to develop self-assembled nanosystem CBSA/siS100A4@Exosome for enhanced drug delivery to cancer cells (Figure 4). The in vivo study demonstrated that CBSA/siS100A4@Exosome has a higher affinity towards cancer cells compared to CBSA/siS100A4@liposomes, and it exhibited significant gene silencing as well as inhibiting the growth of malignant breast cancer cells. The CBSA/siS100A4@Exosome nanoplatform exhibits a promising strategy to impede postoperative metastasis in breast cancer [98,99]. 

Exosomes were collected from fibroblastic BJ cells (BJExo) to treat different cancer cells, including breast tumors [100,101,102,103,104]. *PIK3CA* was silenced by siPIK3CA to improve the breast tumor therapy outcome. *PIK3CA* silencing by siPIK3CA delivery reduced cell migration capacity, impeding the metastasis process. This study confirmed the PI3K/AKT/mTOR pathway and *PIK3CA* as an effective target for breast cancer therapy and introduced BJ-derived exosomes as a promising nanocarrier for siRNA delivery. The study utilized siPIK3CA to target MDA-MB-231 and MDA-MB-453 cell lines and led to the reduction in mRNA expression of *PIK3CA, AKT*, and *mTOR* [104]. The study outcome was supported by previous results [101,102,103]. 

Exosome-loaded siRNA therapeutics have been effective in downregulating BCL-2, PLK1, KRAS, and survivin anti-apoptotic proteins that induce tumor cell mitosis and cell growth factor. The exosome-mediated siRNA therapy was effective in inhibiting cell proliferation and migration. BCL-2 siRNA-loaded exosome derived from NK cells was utilized to treat ER+ breast cancer cells and led to increased apoptosis in breast cancer cells [105]. 

RNA-NP-modified exosome was fabricated to target three cancer cells, including breast cancer cells, simultaneously. In the structure of the RNA-NP, the pRNA of phage phi29 was extended into an arrow shape to connect with the RNA ligands, and fluorescent alexa647 was added for imaging [106]. The RNA ligand was utilized for specific targeting and exosome for effective membrane fusion. The resulting ligand-displaying exosome was capable of specific delivery of siRNA to cells and potentially inhibiting tumor growth. The exosome harboring EGFR aptamer-loaded siSurvivin NPs (EGFRapt/Exosome/siSurvivin) inhibited breast tumor in MDA-MB-468 cells orthotopic xenograft breast tumor bearing mice [106,107]. 

Ligands can be expressed, fusing with exosomal surface proteins by engineered exosome-producing cells, for targeting cancer cells [108]. Modified exosome-producing engineered HEK293T cells were utilized to target HER2+ breast cancer cells. The result demonstrated that HEK293T cells formed DARPin G3 on the surface of exosomes. These modified exosomes can bind to HER2/neu and deliver siRNA to SKBR3 breast cancer cells, downregulating gene expression up to 70%. This study exhibited a potential approach to facilitate gene and drug transfer to HER2+ cancer cells [108]. 

MCF-7 breast cancer cell-derived exosomes loaded with siRNA were used against CD44 in breast cancer cells. Exosome-siRNA targeting CD44 expression (Exos-siCD44) effectively silenced the expression. When cocultured on Exos-siCD44, breast cancer cells demonstrated diminished cell proliferation and improved sensitivity to doxorubicin. The same effect was detected in vivo in mice [109]. 

Nanoscale exosome-mimics (Ems) were generated from non-tumorigenic MCF-10A cells, and then siRNA was encapsulated into the EMs by the electroporation technique. The CDK4siRNA-loaded EMs significantly downregulated CDK4 mRNA protein expression in vitro and in vivo. Therefore, EMs from similar cell types may be effective as siRNA carriers for the development of innovative breast cancer therapeutics with no toxicity [110]. There have been numerous pre-clinical studies utilizing biopolymer-based siRNA delivery systems with promising results for breast cancer therapy (Table 2). Biopolymer-induced siRNA delivery is a prospective strategy to treat breast tumors to improve survival of breast cancer patients.

## 5. Conclusions and Future Perspective

Biopolymers have been proficiently applied in siRNA drug delivery to cancer cells, including breast cancer. The sources of the biopolymers are algae, fungi, plants, animals, microbes, etc. Further, exosomes are obtained from tissues, biological fluids, and cells. Biopolymers are effective for the progress of innovative anticancer drug delivery platforms. Biopolymer-based NP systems have been recognized as cost-effective environmentally friendly platforms for effective drug delivery with enhanced selectivity and reduced toxicity. Nanobiopolymers have promising stimuli-responsive properties and therefore can be used to enhance siRNA delivery platforms to undruggable MDR metastatic cancer cells, including breast cancer. The biopolymeric siRNA drug formulations can shield drugs from pH degradation, extracellular trafficking, nontargeted binding sites, and they are accordingly appropriate for drug delivery and internalization into tumors in a controlled release manner. Numerous natural polysaccharide polymers are utilized in siRNA delivery for cancer therapy. The key advantage of polysaccharides is the presence of various functional groups for modification and polymerization. The most frequently applied polysaccharide for siRNA drug delivery to cancer is chitosan, which is biodegradable, biocompatible, inexpensive, and has low toxicity. For increasing the solubility of chitosan and making it suitable for endo-cytoplasmic release, various modifications have been performed. CPP-based siRNA drugs are promising siRNA delivery vehicles, as they can easily cross the cell membrane. Exosome-loaded siRNA therapeutics have been effective in downregulating cancer genes as well as anti-apoptotic and other proteins that prompt tumor cell mitosis and cell growth factors. Exosome-mediated siRNA therapy is promising and prospective in inhibiting cell proliferation and migration in solid tumors, resulting in enhanced tumor inhibition in solid tumors, including breast tumors, improving the survival of patients. However, for producing biopolymer NPs, functionalization and modification should be performed perfectly and precisely for making them pertinent nanocarriers for siRNA-based drug delivery for overcoming systemic and extra-cellular hurdles. Further, before translation of biopolymer-based siRNA drugs into the clinic, their immune activation and toxicity profiles must be addressed effectively.

## Figures and Tables

**Table 1:** siRNA therapeutics in different stages of clinical trials.

siRNA Drugs	Delivery System	Target Gene	Disease	Company	Status/ Phase
Patisiran	LNP-siRNA	TTR	TTR-mediated amyloidosis	Alnylam	Approved, FDA 2018
Givosiran	GalNAc-siRNA	ALAS1	Acute hepatic porphyria	Alnylam	Approved, FDA 2019
Lumasiran	GalNAc-siRNA	HAO1	Primary heperoxaluria type 1	Alnylam	Approved, FDA 2020
Inclisiran	GalNAc-siRNA	PCSK9	Hypercholesterolemia	Alnylam Novartis	Approved, FDA 2021
Vutrisiran (ALN-TTRSCO2)	GalNAc-siRNA	TTR	TTR-mediated amyloidosis	Alnylam	III
Nedosiran (DCR-PHXC)	GalNAc-siRNA	LDHA	Primary heperoxaluria	Alnylam	III
Fitusiran (ALN-AT3SC)	GalNAc-siRNA	AT	Hemophilia A and B and rare blood disorder	Alnylam Sanofi Genzyme	III
Atu027	Liposomal-siRNA, AtuPLEX	PKN3	Solid tumors, advanced or metastatic pancreatic cancer	Silence Therapeutics	II completed
TMK-PLK1	LNP-siRNA	PLK1	Solid tumors, adrenal cortical carcinoma, hepatocellular carcinoma	Arbutus Biopharma	II completed
siG12D LODER	siG12D-LODER™ system and chemotherapy	G12D mutated KRAS	Pancreatic cancer, pancreatic ductal adenocarcinoma	Silenseed Ltd.	II ongoing
ALN-VSP02	LNP-siRNA	VEGF, KSP	Solid tumors	Alnylam	I completed

**Table 2:** Preclinical studies of selected biopolymer-induced siRNA delivery platforms for breast cancer treatment.

Target	Nanoparticle	Cell Line	Reference
RhoA	Chitosan-coated polyisohexylcyanoacrylate (PIHCA) NPs	MDA-MB-231	[111]
Survivin	Lipid substituted polymer	MDA-MB-231	[112]
HB-EGF	Fab’s antibody-modified LNP	MDA-MB-231	[94]
EGFR	Fab conjugated liposomal NPs	MDA-MB-231	[95]
EGFR	PMLA-based nanobioconjugate	MDA-MB-468	[106]
eGFP	F3-targeted liposomal NPs	MDA-MB-231 MDA-MB-435	[113]
VEGF-A, VEGFR-1, VEGFR-2, and neuropilin-1	Chitosan nanocomplexes	Breast tumor induced in rat using N-nitroso-N-methylurea	[114]
Lipocalin-2	ICAM-1 conjugated liposomes	MDA-MB-231	[97]
HR	EpCAM aptamer polyethylineimine	MCF-7	[93]
EGFR	CPP-loaded nanobubbles	MDA-MB-231	[115]
CD44	Exosome	MCF-7	[109]
PIK3CA	BJExo	MDA-MB-231, MDA-MB-453	[104]
SUR, Bcl2	Bovine milk exosomes	MCF-7, MDA-MB-231	[33]
ABCB1	PEG-L-arginine oligo(-alkylaminosiloxane)-PEI polyplex	MCF-7 ADR, MDA-MB-231	[84]

## Data Availability

Not applicable.
